# Complicated pyelonephritis caused by *Proteus alimentorum* in a woman with peritoneal cancer: a case report

**DOI:** 10.1186/s12879-023-08296-8

**Published:** 2023-05-15

**Authors:** Nobuaki Mori, Jun Hirai, Daisuke Sakanashi, Mina Takayama, Akiko Nakamura, Hirotoshi Ohta, Nobuhiro Asai, Hiroshige Mikamo

**Affiliations:** 1 Department of Clinical Infectious Diseases, Aichi Medical University, 1-1 Yazakokarimata , Nagakute- shi, 480-1195 Aichi Japan; 2 Department of Infection Control and Prevention, Aichi Medical University Hospital, 1-1 Yazakokarimata , Nagakute- shi, 480-1195 Aichi Japan

**Keywords:** *Proteus* spp., *Proteus alimentorum*, Pyelonephritis, 16S rRNA gene sequence

## Abstract

**Background:**

*Proteus* spp. are widespread in the environment and comprise a part of the normal flora of the human gastrointestinal tract. Only six species in this genus, including *Proteus mirabilis, Proteus vulgaris, Proteus terrae, Proteus penneri, Proteus hauseri*, and *Proteus faecis*, have been isolated from human clinical specimens. However, there are no reports of *Proteus alimentorum* isolated from humans, and the clinical characteristics of *P. alimentorum* infection are unknown.

**Case presentation:**

An 85-year-old female patient with peritoneal cancer was hospitalized for complicated pyelonephritis and bacteremia caused by *P. alimentorum.* The patient received antimicrobial therapy and was discharged on day 7 of hospitalization. No recurrence was observed 14 days after the treatment. Various methods were used to identify the *Proteus* sp. Furthermore, the VITEK-2 GN ID card resulted in low discrimination between *P. hauseri* and *P. penneri*. Matrix-assisted laser desorption/ionization time-of-flight mass spectrometry showed *P. hauseri* with a spectral score of 2.22 as the best match. Nevertheless, the pathogen was identified as *P. alimentorum* based on genetic investigation using 16 S rRNA gene sequencing and biochemical tests.

**Conclusion:**

*Proteus alimentorum* is a human pathogen, and its infection has an excellent therapeutic response to antimicrobials based on antimicrobial susceptibility. Genomic methods may be helpful for the precise identification of *P. alimentorum*.

## Background

*Proteus* spp. are gram-negative, facultatively anaerobic, short rods with flagella and fimbria and belong to the family *Morganellaceae.* The genus *Proteus* comprises the following nine species: *Proteus mirabilis, Proteus vulgaris, Proteus terrae, Proteus penneri, Proteus hauseri, Proteus faecis, Proteus columbae, Proteus cibi, and Proteus alimentorum* [1]. Furthermore, *Proteus* spp. are widespread in the environment and are a part of the normal flora of the human gastrointestinal tract. Only six species in this genus, including *P. mirabilis, P. vulgaris, P. terrae, P. penneri, P. hauseri*, and *P. faecis*, have been isolated from human clinical specimens [2, 3]. In clinical settings, *Proteus* ranks third as the cause of uncomplicated cystitis, pyelonephritis, and prostatitis [4]. Particularly, *P. mirabilis, P. vulgaris*, and *P. penneri* have been reported as causative agents. Dai et al. found that *P. alimentorum* was isolated from pork and lobsters in 2018 [5]. However, there are no reports of *P. alimentorum* isolated from humans, and the clinical characteristics of *P. alimentorum* infection are unknown.

Here, we report a case of complicated pyelonephritis caused by *P. alimentorum* in a woman with peritoneal cancer.

## Case presentation

An 85-year-old female patient presented to our emergency department with a fever and lower back pain. She had a medical history of diabetes mellitus, and her hemoglobin A1C was at 6.9 with teneligliptin. Additionally, she had undergone bilateral ovariohysterectomy for peritoneal cancer for 2 years and had received molecularly-targeted therapy with olaparib for the last 4 months.

**Fig. 1:**
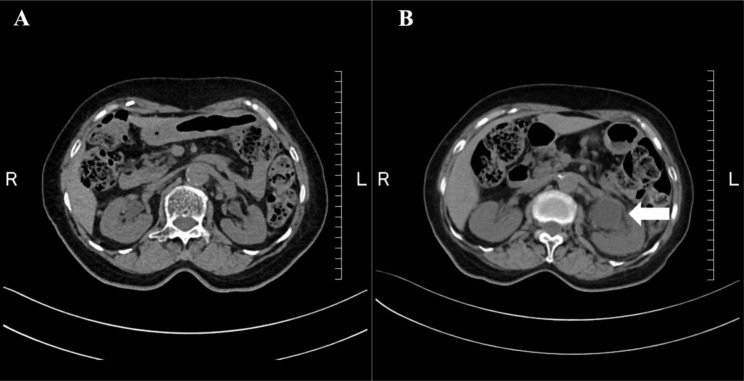
**(A)** An abdominal CT taken one year before admission revealed mild dilation of the left renal pelvis. **(B)** An abdominal CT taken on admission revealed dilation of the left renal pelvis (arrow) with an obstruction of unknown origin, and perinephric stranding of the left renal pelvis was observed CT, computed tomography

Upon examination, the patient was alert and oriented. Her body temperature, blood pressure, pulse, and respiratory rate were 37.4 °C, 122/60 mmHg, 80 beats/min, and 16 breaths/min, respectively. There was no abdominal pain or sign of costovertebral angle tenderness. Laboratory results were as follows: an elevated leukocyte count of 9,800 cells/mm^3^ (normal range: 3,300–8,600), procalcitonin level of 0.18 ng/mL (normal range: ≤0.05), C-reactive protein level of 3.75 mg/dL (normal range: ≤0.04), and slightly elevated serum creatine level of 1.0 mg/dL (normal range: 0.46–0.79). Urinalysis revealed turbid urine with an alkaline pH (8.5), occult blood, white blood cells, and nitrate. Computed tomography revealed dilation of the left renal pelvis with an obstruction of unknown origin and perinephric stranding of the left renal pelvis but no evidence of renal stones (Fig. 1). On the second day, two sets of blood and urine cultures obtained on admission revealed gram-negative rods. Ceftriaxone (1 g every 12 h) was administered since acute pyelonephritis was considered the diagnosis. The VITEK-2 GN ID card (bioMerieux, France) resulted in low discrimination between *P. hauseri* and *P. penneri*. Matrix-assisted laser desorption/ionization time-of-flight mass spectrometry (MALDI-TOF MS; MALDI Biotyper ver. 9.0.0.0; Bruker Daltonics, Billerica, MA, USA) showed *P. hauseri* with a spectral score of 2.22 as the best match and *P. vulgaris* with a score of 2.09 as the second-best match. Therefore, the genetic investigation by 16 S rRNA gene sequencing using the forward primer 5′-AGAGTTTGATCMTGGCTCAG-3′ and the reverse primer 5′-TACGGYTACCTTGTTACGACTT-3′ was performed to identify the organism. Finally, the pathogen was identified as *P. alimentorum* with 99.8% homology (1,473 of the 1,476 bases) in the EZBioCloud 16 S database (http://www.ezbiocloud.net/eztaxon). To confirm that the biochemical properties of the isolate were consistent with those of *P. alimentorum*, palatinose utilization and indole production were investigated to differentiate the isolate from *P. hauseri, P. vulgaris*, and *P. penneri* (Table 1). The isolate showed positive results for palatinose utilization and indole production, consistent with those of a previous report on *P. alimentorum*. The GenBank accession number of the 16SrRNA sequences of *P. alimentorum* isolated from this study is OQ192985. **Table 2** shows the antimicrobial susceptibility of the isolated strain determined using the MicroScan WalkAway system with an NM2J panel (Beckman Coulter). The minimum inhibitory concentration was measured according to the *Enterobacteriaceae* category of the Clinical and Laboratory Standards Institute M100-S26.

**Table 1:** Biochemical characteristics of the isolate and Proteus spp

	Indole production	Palatinose utilization
*Proteus alimentorum*	+	+
*Proteus hauseri*	+	-
*Proteus penneri*	-	+
*Proteus vulgaris*	+	-
Isolate	+	+

Antimicrobial therapy was changed to cefotiam (1 g every 8 h) based on the antimicrobial susceptibility results (Table 2). As the patient’s clinical course was good, the antimicrobial therapy was changed to ciprofloxacin (400 mg orally), and she was discharged on day 7 of hospitalization. Antimicrobial therapy was continued for 14 days, and no recurrence was observed.

**Table 2:** Antimicrobial susceptibilities of the isolate

Antibiotics	Minimum Inhibitory Concentration (μg/mL)	Interpretation*
Ampicillin	16	I
Cefazolin	> 16	R
Cefotiam	≤ 2	S
Cefotaxime	≤ 1	S
Ceftazidime	≤ 1	S
Cefepime	≤ 2	S
Aztreonam	≤ 1	S
Cefmetazole	≤ 4	S
Latamoxef	≤ 2	S
Imipenem	2	I
Dripenem	≤ 1	S
Meropenem	≤ 0.125	S
Sulbactam/Ampicillin	≤ 8/4	S
Tazobactam/piperacillin	≤ 4/4	S
Amikacin	≤ 4	S
Minocycline	≤ 4	S
Levofloxacin	≤ 0.125	S
Sulfamethoaxazole/trimethoprim	≤ 2/38	S
Fosfomycin	≤ 64	S

Abbreviation: S, susceptible; I, intermediate; R, resistant

*The interpretation criteria as recommended by the Clinical and Laboratory Standards Institute M100-S26

## Discussion and conclusions

Here, we describe a case of complicated pyelonephritis caused by *P. alimentorum* in a woman with peritoneal cancer. Although it is difficult to identify the isolates as *P. alimentorum* using MALDI-TOF MS and conventional methods, 16 S rRNA gene sequencing analysis and biochemical property tests are useful. We thoroughly searched various databases, including PubMed, Google Scholar, CINHAL, MEDLINE (EBSCOhost), and Web of Science, for *Proteus alimentorum* infection cases. However, we found no reports of *P. alimentorum* infections in humans. To the best of our knowledge, this is the first documented clinical case report of a *P. alimentorum* infection in a human.

*Proteus alimentorum* was identified as a novel species of the genus *Proteus* in 2018 [5]. This was isolated from pork and lobster during an investigation of food poisoning in Maanshan, Anhui Province, China, in 2008 (the causal relationship between this bacterium and food poisoning is unknown) [5]. This bacterium was identified as a new species distinct from the traditional Proteus by a polymorphic taxonomic study that included phenotypic, phylotypic, and genotypic methods. This organism is an indole-positive *Proteus* sp. and a gram-negative, facultatively anaerobic, short-rod bacterium that is motile owing to its flagellum. Our isolate showed that both *P. hauseri* and *P. vulgaris* scored high on the Bruker MALDI Biotyper. It is usually impossible to accurately determine the organism in the case of multiple bacterial species reported with a score of 2 or more by the Bruker MALDI Biotyper. To accurately identify organisms,16 S rRNA gene sequencing should be performed. Several biochemical characteristics can be used to distinguish between these organisms [4]. *Proteus alimentorum* and *P. hauseri* swarm on 1.5% agar, but *P. vulgaris* does not. *Proteus alimentorum* is positive for arbutin oxidation, aesculin hydrolysis, and salicin fermentation and utilizes palatinose, whereas *P. hauseri* is negative for them and cannot utilize palatinose. *Proteus alimentorum* utilizes palatinose, tyrosine, and α-glucosidase, whereas *P. vulgaris* cannot. Palatinose utilization and indole production were useful for determining the biochemical properties of *P. hauseri, P. penneri*, and *P. vulgaris*, which differed from *P. alimentorum* in VITEK-2 and the Bruker MALDI Biotyper in this study.

*Proteus* spp. cause various infections that range from uncomplicated urinary tract infections (UTIs) to life-threatening infections of the abdomen, skin, soft tissue, lung, and other sites in both immunocompetent and immunocompromised hosts, with UTIs being the most common [2]. We diagnosed *P. alimentorum* as the causative microorganism of UTI. It was isolated from the urine, and two sets of blood cultures and computed tomography findings suggested inflammation of the left kidney. Additionally, urine examination revealed an alkaline pH, which may be due to the production of urease and the splitting of urea into NH_3_ and CO_2_ by *Proteus*. Most *Proteus* spp. are susceptible to β-lactam antibiotics; however, *P. vulgaris* and *P. penneri* produce chromosomally encoded inducible class A cefuroximase, conferring resistance to penicillin and first- and second-generation cephalosporins [2, 6]. Dai et al. examined the antimicrobial susceptibility of *P. alimentorum* to gentamicin, ceftriaxone, imipenem, kanamycin, sulfisoxazole, cefoxitin, cefepime, ciprofloxacin, streptomycin, sulfamethoxazole, nalidixic acid, doxycycline, chloramphenicol, tetracycline, ampicillin, and azithromycin. *Proteus alimentorum* showed sensitivity to all antibiotics except for intermediate resistance to ampicillin and resistance to azithromycin [5]. Our isolate showed intermediate resistance to ampicillin and imipenem and resistance to cefazolin. We treated the patient with second- and third-generation cephalosporins and fluoroquinolones with good clinical outcomes. Antimicrobials should be selected based on antimicrobial susceptibility.

This report showed that *P. alimentorum* is a pathogen in humans. *Proteus alimentorum* infection showed a good therapeutic response to antimicrobials based on antimicrobial susceptibility. Genomic methods may be useful for the precise identification of *P. alimentorum*.

## Data Availability

The datasets analyzed during the current study are available in the GenBank repository (accession number: OQ192985)(URL: https://www.ncbi.nlm.nih.gov/nuccore/OQ192985).

## Abbreviations

MALDI-TOF MS: Matrix-assisted laser desorption/ionization time-of-flight mass spectrometry
UTIs: urinary tract infections
